# The structure of legume–rhizobium interaction networks and their response to tree invasions

**DOI:** 10.1093/aobpla/plw038

**Published:** 2016-07-11

**Authors:** Johannes J. Le Roux, Natasha R. Mavengere, Allan G. Ellis

**Affiliations:** Department of Botany and Zoology, Centre for Invasion Biology, Stellenbosch University, Matieland, 7602, South Africa

**Keywords:** Biological invasions, co-introduction, cosmopolitan rhizobia, legume–rhizobium interaction webs, network specialization

## Abstract

Establishing mutualistic interactions in novel environments is important for the successful establishment of some non-native plant species. These associations may, in turn, impact native species interaction networks as non-natives become dominant in their new environments. Using phylogenetic and ecological interaction network approaches we provide the first report of the structure of belowground legume–rhizobium interaction networks and how they change along a gradient of invasion (uninvaded, semi invaded and heavily invaded sites) by Australian *Acacia* species in South Africa’s Cape Floristic Region. We found that native and invasive legumes interact with distinct rhizobial lineages, most likely due to phylogenetic uniqueness of native and invasive host plants. Moreover, legume–rhizobium interaction networks are not nested, but significantly modular with high levels of specialization possibly as a result of legume–rhizobium co-evolution. Although network topology remained constant across the invasion gradient, composition of bacterial communities associated with native legumes changed dramatically as acacias increasingly dominated the landscape. In stark contrast to aboveground interaction networks (e.g. pollination and seed dispersal) we show that invasive legumes do not infiltrate existing native legume–rhizobium networks but rather form novel modules. This absence of mutualist overlap between native and invasive legumes suggests the importance of co-invading rhizobium–acacia species complexes for *Acacia* invasion success, and argues against a ubiquitous role for the formation and evolutionary refinement of novel interactions.

## Abstract (precis)

We provide data on how legume-rhizobia interaction webs react to invasions by exotic legumes. This is the first study of its kind and found that general hypotheses derived from above-ground mutualistic webs may not hold for below-ground counterparts. Specifically, we found that legume-rhizobia interactions at the community level are highly specialised resulting in strongly modular webs, which are not nested, and that invasive legumes do not infiltrate existing native webs but rather form unique and novel modules in webs.

## Introduction

Invasive species can change the composition, structure, and functioning of communities they invade, and therefore the way species interact (Vitousek 1990). Numerous studies have shown the impacts of invasive species on the interactions between native plants and their mutualists; such as pollinators (e.g. Aizen *et al.* 2012; Bartomeus *et al.* 2010; Vilà *et al.* 2009) and seed dispersers (e.g. Spotswood *et al.* 2012; Heleno *et al.* 2013). From these, two oversimplified generalizations have emerged - that invasive plant species are often generalized in their mutualistic requirements (Bascompte, 2009) and that this allows them to utilize existing mutualists found in their new ranges through interaction web infiltration (e.g. Bascompte 2009; Bartomeus *et al.* 2010; Aizen *et al.* 2012). Mutualistic interactions usually span a continuum, from being generalized to highly specialized, leading to the expectation of nested interaction network structure, i.e. specialists in the community will only interact with a subset of the species that generalists interact with (Bascompte, 2009). Specialization also means that any pair of species will not necessarily have the same probability of interacting and therefore groups of host plants may only interact with a given group of co-evolved mutualists, and *vice versa*, i.e. network modularity (Bascompte, 2009). Thus, the emergent structure (i.e. modular or nested) of interaction networks likely depends on the distribution of interaction specialization within the interacting communities. For example, for pollination interactions, where plants have limited control of pollinator foraging, we might expect a range of interaction specialization and therefore nested network structures (Bascompte *et al.* 2006). In contrast, in functionally critical plant-microbe mutualisms such as between legumes and rhizobia, the focus of this paper, where pairwise species interactions are often controlled by gene-for-gene interactions (Spaink 2000), high levels of co-evolved specialization might be expected and hence strong network modularity. This, coupled with the known impacts of plant invasions on soil microbial community composition and function (for review see Bissett *et al.* 2013), makes legume–rhizobium associations a fascinating system to gain additional insights into the impacts of invasions on mutualistic interaction network structures. To our knowledge no studies have explored legume-rhizobium networks to date.

Rhizobia are bacteria capable of entering their legume hosts through root hairs, sites of lateral root emergence, or directly through root epidermis (Sprent *et al.* 2013), where they can induce the development of nodules where biological nitrogen fixation takes place (Gage 2004). The formation of root nodules involves complex molecular signalling pathways between legumes and rhizobia (Stacey 2007). Various bacterial nodulation genes (so-called Nod genes) respond to plant root exudates (typically flavonoids) by producing nodulating factors (nod factors), leading to the initiation of root nodule formation. These nod factors are thought to be important determinants of legume–rhizobium specificity (Spaink 2000). Nod genes are located on symbiotic plasmids or symbiotic islands (Rogel *et al.* 2011), highly mobile genetic elements that can be transferred between different rhizobial species and even genera by horizontal gene transfer (HGT) (Ding and Hynes 2009). Within root nodules, organic forms of reduced atmospheric nitrogen produced by the bacteria are utilized by the host plant and ultimately enter the earth’s food webs. In exchange, bacterial symbionts acquire carbohydrates from legumes. Surprisingly, rhizobia are not monophyletic and represent a diverse array of bacteria found in both the Alphaproteobacteria (‘alpha rhizobia’, e.g. genera *Rhizobium* and *Bradyrhizobium*) and Betaproteobacteria (‘beta rhizobia’, e.g. genera *Burkholderia* and *Cupriavidu*s) classes (Gyaneshwar *et al.* 2011).

The unique partnership between legumes and rhizobia has been suggested as a major contributing factor to the success of some legumes as prominent invasive species in many parts of the world (Parker 2001) and that the ability to find ‘compatible’ rhizobia in introduced regions plays an important role in establishment success of legumes (Rodríguez-Echeverría *et al.* 2011). Two scenarios are plausible, either invasive legumes form interactions with native rhizobia, or they are co-introduced with their symbionts. The former would be facilitated by generalism in invasive legumes (i.e. increasing the probability of encountering suitable rhizobia in the invasive range) or by evolutionary change allowing establishment of novel interactions with native rhizobia. Novel associations and co-introductions of legumes and rhizobia should result in very different interaction network signatures. Under co-invasion, the introduced legume–rhizobium partnership should form an isolated module within the interaction network; while under the alternative scenario we would expect overlap in rhizobial partners between native and invasive legumes (i.e. they should be connected by shared rhizobia). Invasive trees in the Australian legume genus *Acacia* Mill. *sensu stricto* (Leguminosae subfam. Mimosoideae, formerly *Acacia* subgen. Phyllodineae DC; Maslin 2008), have received much research attention because of their invasion success and severe impacts on native ecosystems globally (Richardson *et al.* 2011). Acacias are known to form successful rhizobial interactions in their introduced ranges (e.g. Birnbaum *et al.* 2012; Wandrag *et al.* 2013) and have, in some instances, been co-introduced with their rhizobia (Crisóstomo *et al.* 2013; Ndlovu *et al.* 2013; Rodríguez-Echeverría, 2010). However, whether they integrate into, and impact on, native community interaction networks remains unknown.

Here, employing phylogenetic and network approaches across a gradient of invasion (uninvaded, semi-invaded [invasion periphery] and heavily invaded [invasion core]) in South Africa’s Cape Floristic Region (CFR), we elucidate interaction networks between invasive acacias and native legumes and their associated root nodule bacteria. Specifically we address three questions: (i) First we test the hypothesis that legume–rhizobium networks are structured by strong co-evolved relationships, in which case we expect interaction networks to be strongly modular. (ii) Alternatively, we test the expectation that invasive legumes should infiltrate native legume–rhizobium networks (i.e. exhibit substantial overlap in interactions with natives) under the hypothesis that invasive legumes form or evolve associations with the available native rhizobium community. If legume invasion involves co-introduction of legume–rhizobium species complexes we expect that invasive legumes will not integrate into native networks, but will instead form novel network modules. (iii) Finally we test for changes in rhizobial associations of native legumes at invaded sites, which would be the expectation if invasive legumes compete for interaction partners with native legumes, or if co-invading bacteria interact with native legumes and/or rhizobial communities.

## Methods

### Study site and field collections

**Figure 1.**
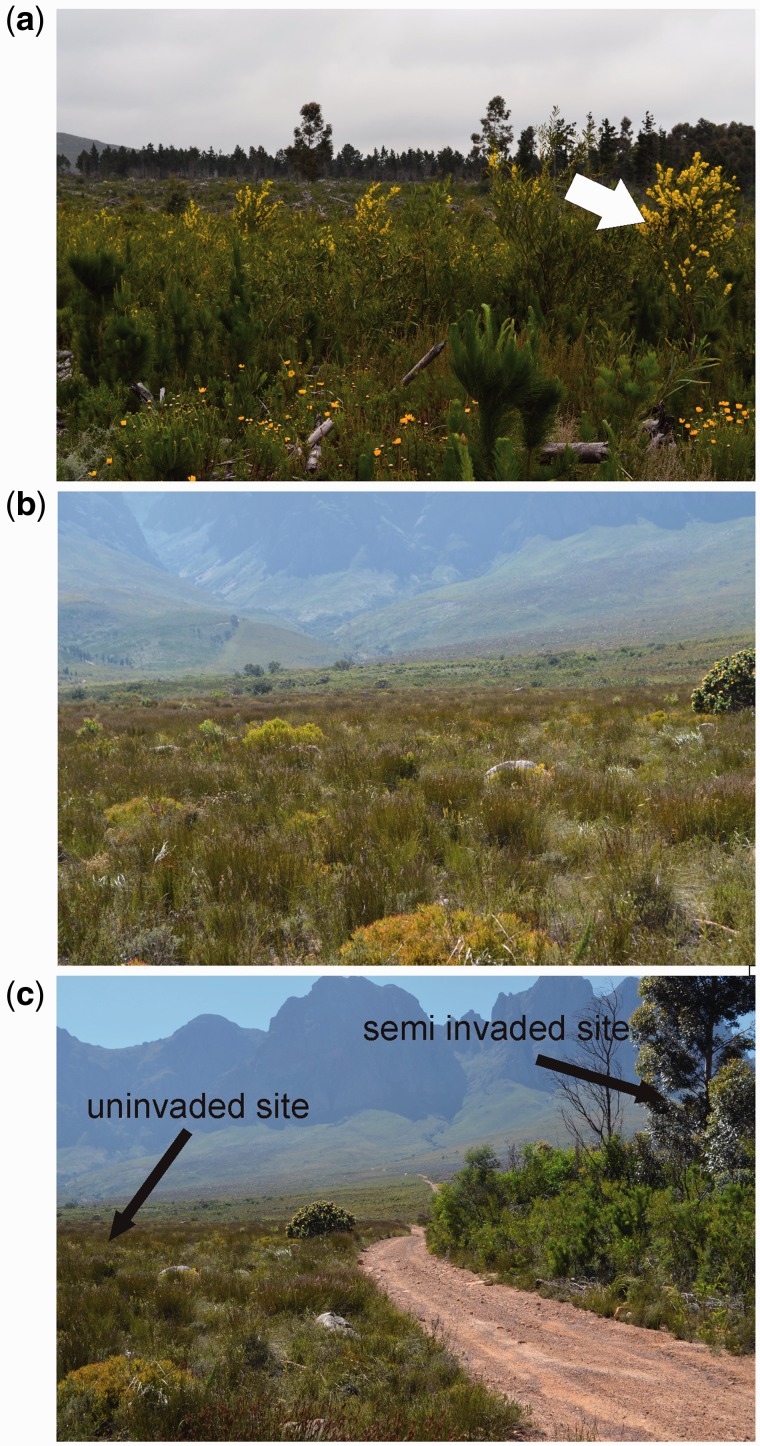
Photographs illustrating semi invaded **(a)** and univaded **(b)** sites in close proximity to each other **(c)** at Helderberg region that were sampled for rhizobia and legumes in this study, showing the presence (white arrow) of acacias that were absent from the nearby univaded site (Photos by JJ Le Roux).

Our study area, in the Helderberg area of South Africa’s Western Cape Province, represented a gradient of invasion (uninvaded site [never invaded by Australian acacias], semi-invaded site [invasion periphery, 1–10 acacias/m^2^] and heavily invaded site [invasion core, >10 acacias/m^2^]; Fig. 1). Heavily invaded sites not only had higher tree densities but also had older trees compared with the semi-invaded area. All sites fell within a 600 m transect. All native legumes were sampled and herbarium samples submitted to the Compton Herbarium (Cape Town, South Africa) for expert identification. Root nodules were also sampled from all native and invasive legume species present at all three sites. At each site ten root nodules from at least five individuals per species were collected, dehydrated and kept on silica gel until needed for bacterial isolations [**see Supporting Information**].

### Rhizobial isolation, culturing and identification

Root nodules were rehydrated, surface sterilized and rhizobia isolated and cultured following protocols described by Somasegaran and Hoben (1994) with minor modifications; the sterilization step in acid was omitted and nodules were washed for 60 s in 3.5% sodium hypochlorite instead. Pure colonies were picked and used in colony PCR. For bacterial identification, partial 16S rRNA gene regions were amplified and sequenced using the primers E9F (Farrelly *et al.* 1995), 341F (Muyzer *et al.* 1993), 536R and 907F (Lane *et al.* 1985), 1100R (Lane 1991) and 1512R (Weisburg *et al.* 1991). Each 50 µL PCR reaction contained a final concentration of 200 µM of each dNTP (AB gene, supplied by Southern Cross Biotechnologies, Cape Town, South Africa), 25 pmoles of each primer, 5 U Taq DNA polymerase (Super-Therm JMR-801, Southern Cross Biotechnologies, Cape Town, South Africa), 1 × PCR reaction buffer, 1.5 mM MgCl_2_ and 10 µL of bacterial colony suspension in water. PCR followed a cycle of initial denaturation of 95 °C for 5 min; 35 cycles at denaturation at 94 °C for 30 s, annealing at 58 °C for 60 s, elongation at 72 °C for 90 s; and final extension at 72 °C for 10 min. PCR products were purified using the QIAquick PCR Purification Kit (Qiagen, supplied by Whitehead Scientific, Cape Town, South Africa) and sequenced using the ABI PRISM BigDye Terminator Cycle Sequencing Ready Reaction kit and an automated ABI PRISM 377XL DNA sequencer (PE Applied Biosystems, Foster City, CA, USA).

Sequences were edited using BIOEDIT version 7.0.5.3 (Hall, 1999). Edited sequences were blasted against GenBank accessions (http://blast.ncbi.nlm.nih.gov/Blast.cgi.) and the EzTaxon-e server (http://eztaxon-e.ezbiocloud.net/; Kim *et al.* 2012) to determine sequence similarity and identity against known rhizobia species. Only taxa known from the literature to form nitrogen-fixing symbiosis with legumes, including potentially novel species, were retained for further analyses.

### Phylogenetic analysis

Identification revealed that we isolated both alpha and beta rhizobia. Given the evolutionary distances between these two classes we created separate data partitions for each. We also included data for reference strains that closely resembled our rhizobia based on identifications as described earlier. Australian acacias are predominantly nodulated by *Bradyrhizobium* spp. (Rodríguez-Echeverría *et al.* 2011). We therefore also included previously published *Bradyrhizobium* strains isolated from legumes, including acacias, from Australia. For both partitions (alpha and beta rhizobia) nearly-full length 16S rRNA gene sequences were aligned using MAFFT (Katoh *et al.* 2002) with manual adjustments in BIOEDIT version 7.0.5.3 (Hall, 1999).

We reconstructed phylogenies based on maximum likelihood search criteria in Mega version 6.0 (Tamura *et al.* 2013). Akaike scores from jModeltest v 2.1.4 identified the best-fit model as the TrN + G and GTR + I + G (determined using jModelTest v. 2, Darriba *et al.* 2012) for the alpha and beta rhizobia datasets, respectively. Nodal support for retrieved tree topologies was calculated as bootstrap values.

### Nodule occupancy and legume–rhizobium interaction networks

Quantitative legume–rhizobium interaction matrices were constructed for each site across the invasion gradient and for the combined dataset using the frequency of nodule occupancy by bacterial taxa as the interaction weights. Between 18 and 20 bacterial 16S rRNA DNA sequences were available for each of the five legume species sampled at each site (totals per site: uninvaded—100, semi-invaded—97, heavily invaded—99 sequences). Given uncertainty for optimal 16S rRNA gene sequence similarity within and between bacterial species (Kim *et al.* 2014), and in order to test how bacterial phylogenetic relatedness impacted our network results, we performed network analyses on hierarchical datasets representing genotypes and clusters of sequences (‘bacterial lineages’) at 99, 98 and 95% DNA sequence similarity levels. This approach allowed us to gauge the extent to which bacterial taxon delineation influenced our inferences about network structure.

We used the bipartite package version 2.01 in R (Dormann *et al.* 2009) to calculate eight metrics to describe network topology: connectance (I/P*B: the proportion of possible links observed in a web, where I describes the number of realized links, and P and B represent number of interacting plant species and rhizobial strains respectively), interaction evenness (IE = H/*ln* L: based on Shannon diversity (H), where L is the number of all links, Tylianakis *et al.* 2007), weighted nestedness (WNODF: degree based interaction frequencies indicating the extent to which species with few links in a bipartite network represent a subset of the links of other species, e.g. specialists interacting with only a subset of generalist partners, Almeida-Neto and Ulrich, 2011), weighted modularity (QuanBiMod—Q: aggregates of interacting species where groups of legumes and rhizobia share interactions more frequently within modules than across modules, Dormann and Strauss 2014), number of identified modules, network specialization (H’_2_: the link complementarity across all species, where high specialization indicates high dependency of each species on a few exclusive partners and low specialization indicates higher functional redundancy, Blüthgen *et al.* 2007) and generality (weighted mean number of effective partners: Bersier *et al.* 2002) of plants (Gp) and bacteria (Gb). The significance of observed nestedness was determined by comparison with sets of 10^3^ randomized networks generated keeping marginal totals in the network fixed. Weighted modularity of each network was determined as the highest *Q* value from five independent runs of the algorithm, each terminated after 10^6^ swaps. Significance was assessed by converting observed *Q* values to *z*-scores indicating departure from the expected mean modularity scores of 100 randomized networks, generated keeping marginal totals in the network fixed. In addition we calculated three species level network metrics for plants and bacteria to further explore specialization of taxa across sites: degree (counts of numbers of connections), number of effective partners (Bersier *et al.* 2002) and specialization (d’: a measure of the exclusivity of interactions that individual species take part in, Blüthgen *et al.* 2006). These metrics were compared (i) across sites and (ii) between native and invasive legumes using ANOVA or Kruskal-Wallis depending on whether data were parametric. For comparisons between natives and invasives we averaged metrics for species that occurred at more than one site prior to analysis.

**Figure 2.**
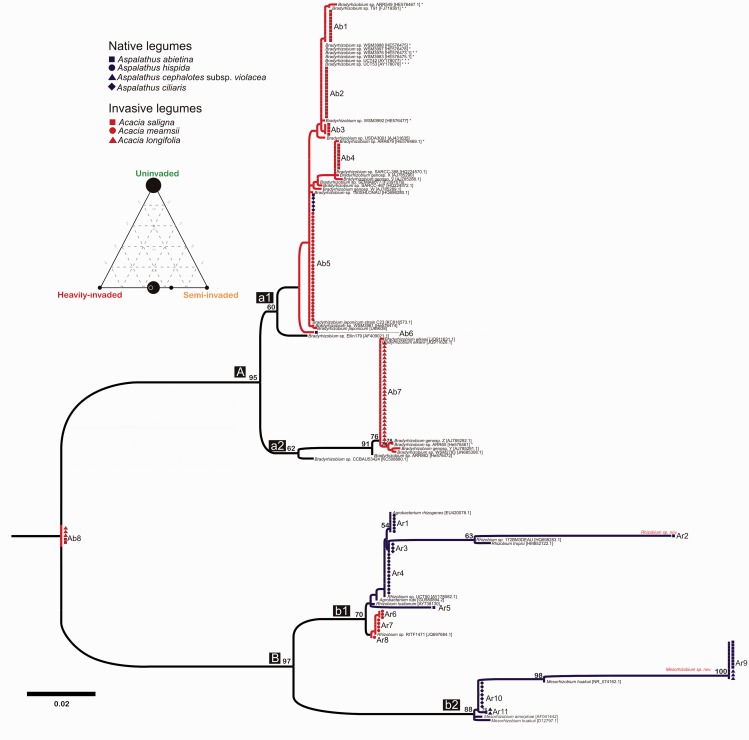
Phylogeny based on partial 16S rDNA sequences for rhizobia taxa belonging to the alphaproteobacteria subclass. Blue branches indicate collections from legumes at uninvaded sites and red branches subtend those collected from semi and heavily invaded sites. Different symbols and colours at tips correspond to different host plants. Representative taxa from GenBank were included for phylogeny reconstruction with taxon names indicated in black. * indicate *Bradyrhizobium* spp. previously isolated from Australian legumes, ** those isolated from acacias and *** those isolated from CFR legumes. Putative novel species based on DNA distances to previously described species are shown in red above branches. Support for tree topology is given as bootstrap values (>50) at nodes. The inserted ternary diagram illustrates the relative frequency of occurrence of individual bacterial genotypes (black circles) across the three sites where the size of each circle corresponds to the number of bacterial 16S rDNA genotypes. Note that no genotypes were shared between the uninvaded and invaded (semi and heavily invaded) sites, whereas most genotypes present at the semi-invaded site were also present in similar frequencies at the heavily invaded site.

Next we explored the frequency of nodule occupancy by major bacterial clades (i.e. alpha rhizobia—slow-growing *Bradyrhizobium* clade (Fig. 1, clade A), alpha rhizobia—fast-growing ‘*Rhizobium*’ clade (Fig. 1, clade B), beta rhizobia—*Burkholderia* spp. (Fig. 2)) across sites and between native and invasive legumes. Data from the partially and heavily invaded sites were combined for these analyses as our networks revealed very similar bacterial communities and interactions. We used G-tests to ask (i) whether differences in the frequency of nodule occupancy by bacteria in these major clades differs between invasive and native legumes at the invaded sites and (ii) whether composition of nodule occupants changes across the invasion gradient in the native species present at uninvaded and invaded sites (i.e. for *Aspalathus abietina* and *Aspalathus* *ciliaris*).

## Results

### Rhizobial isolation, culturing and identification

**Figure 3.**
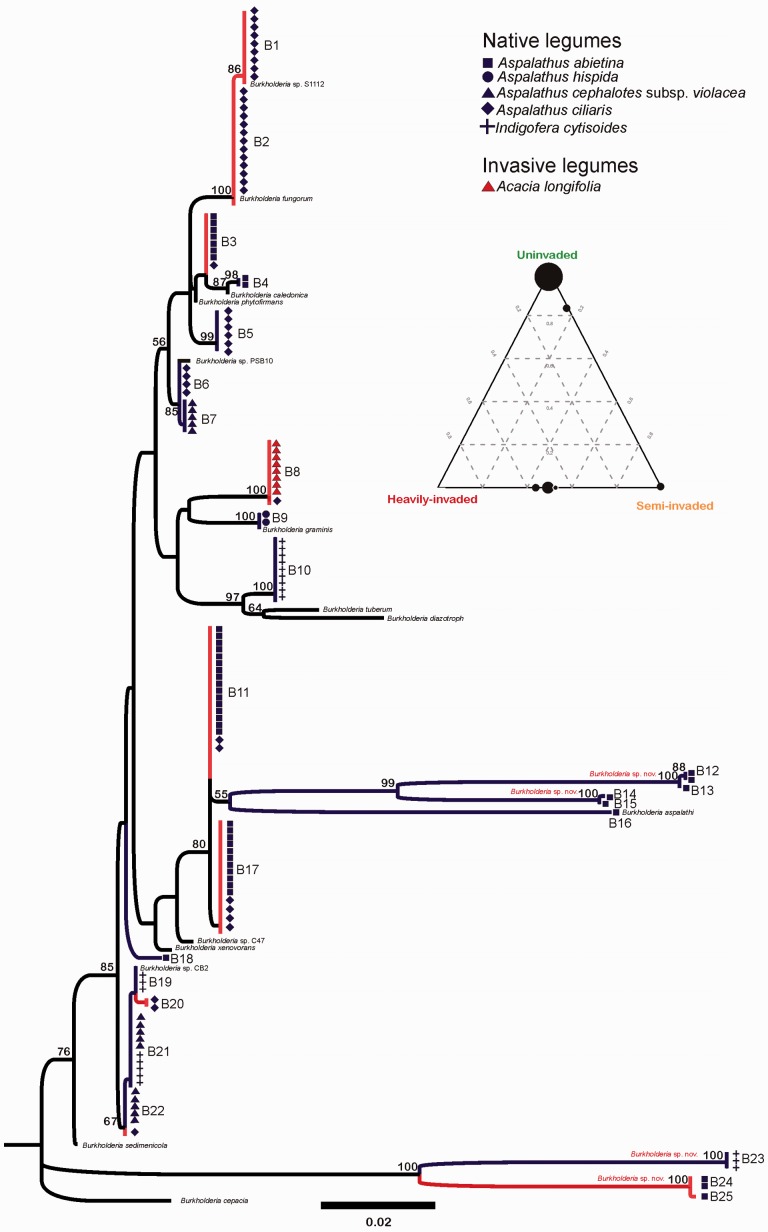
Phylogeny based on partial 16S rDNA sequences for rhizobia taxa belonging to the betaproteobacteria subclass within the genus *Burkholderia*. Blue branches indicate collections from legumes at uninvaded sites and red branches subtend those collected from semi and heavily invaded sites. Different symbols and colours at tips correspond to different host plants. Representative taxa from GenBank were included for phylogeny reconstruction. Putative novel species based on DNA distances to previously described species are shown in red above branches. Support for tree topology is given as bootstrap values (>50) at nodes. The inserted ternary diagram illustrates the relative frequency of occurrence of individual bacterial genotypes (black circles) across the three sites where the size of each circle corresponds to the number of bacterial 16S rDNA genotypes. Note that no genotypes were shared between the uninvaded and heavily invaded and one between semi invaded and uninvaded sites, whereas most genotypes present at the semi-invaded site were also present in similar frequencies at the heavily invaded site.

A total of five native fynbos legumes were identified (*A.* *abietina* Thunb., *Aspalathus cephalotes* Thunb., *A.* *ciliaris* L., *Aspalathus hispida* Thunb., subsp. *violacea*, and *Indigofera cytisoides* (L.) L.), all of which were present in the uninvaded site. *A.* *abietina* and *A. ciliaris* were the only native species present in the semi and heavily invaded sites. Invasive *Acacia mearnsii*, *Acacia* *longifolia* and *Acacia* *saligna* were all present at the semi and heavily invaded sites, but the heavily invaded site was dominated by *A. mearnsii*. Root nodules were present on all native and invasive legumes. Identification using 16S rRNA gene sequences revealed a high diversity of alpha and beta rhizobia associated with both native and alien legumes (Figs. 2 and 3). The majority of alpha rhizobia associated with invasive acacias represented slow-growing *Bradyrhizobium* spp. (86% of nodule occupants—Table 1, Fig. 2). In contrast, native legumes across all sites were predominantly associated with beta rhizobia (Table 1, Fig. 3), but also formed associations with fast-growing alpha rhizobia, representing strains of *Rhizobium* and *Mesorhizobium*, at the uninvaded site. Low sequence similarity (97%, following Kim *et al.* 2014) between some isolates and accessions from Genbank suggested that novel and currently undescribed species from the genera *Bradyrhizobium*, *Burkholderia, Rhizobium* and *Mesorhizobium* were associated with *A.* *abietina, A. cephalotes* subsp. *violacea* and *I.* *cytisoides* (Figs. 2 and 3), one of which was subsequently described as *Burkholderia aspalathi* (Mavengere *et al.* 2014).

**Table 1.** Percentage nodule occupancy of major bacterial clades in five native and three exotic legume species at invaded (semi and heavily) and uninvaded sites. Major clade level composition of nodule occupying bacteria differed significantly between native and invasive legumes at the invaded sites (*G* = 154.7, df = 2, *P* < 0.0001) and between uninvaded and invaded sites for the natives *A. abietina* and *A. ciliaris* (*G* = 44.2, df = 2, *P* < 0.0001). Alpha rhizobia in the ‘Rhizobium’ clade (see Fig 2., clade B) were virtually absent from invaded sites.

		**Uninvaded**			**Invaded**				
		Beta (*Burkholderia*)	Alpha (*Rhizobium*)	Alpha (*Bradyrhizobium*)	Beta (*Burkholderia*)	Alpha (*Rhizobium*)	Alpha (*Bradyrhizobium*)
Invasive	*A. longifolia*				21	0	79
	*A. saligna*				0	0	100
	*A. mearnsii*				0	20	80
	**ALL**				**7**	**7**	**86**
Native	*A. abietina*	45	55	0	95	0	5
	*A. ciliaris*	50	50	0	89	0	11
	*A.* *cephalotes*	75	25	0			
	*A. hispida*	10	90	0			
	*I. cytisoides*	100	0	0			
	**ALL**	**56**	**44**	**0**	**92**	**0**	**8**

### Phylogenetic analysis

The aligned alpha rhizobia partial 16S rRNA matrix contained 950 base pairs (bp), and 17 gaps (indels) that were between one and seven bp in size. The beta rhizobia partial 16S rRNA matrix contained 963 bp and 15 indels between one and ten bp in size. All DNA sequences have been deposited in GenBank (accession numbers KU836936-KU837225, http://www.ncbi.nlm.nih.gov). The alpha rhizobia phylogeny retrieved two distinct and well-supported clades, one containing slow-growing *Bradyrhizobium* spp. (Fig. 2, clade A) and another containing fast-growing *Agrobacterium*, *Mesorhizobium* and *Rhizobium* spp.(Fig. 2, clade B). Overall, these two clades corresponded mostly to alien (clade A) and native (clade B) legume-associated rhizobia. Interestingly, different invasive *Acacia* spp. associated with phylogenetically distinct rhizobia: *A. mearnsii*- and *A. saligna*-associated rhizobia clustered with lineages closely related to *Bradyrhizobium japonicum* (Fig. 2, node a1), while *A. longifolia* was associated with strains closely related to *Bradyrhizobium elkanii* (Fig. 2, node a2). Beta rhizobia from the genus *Burkholderia* were almost exclusively associated with indigenous legumes (Fig. 3). *Acacia* *longifolia* was the only alien species from which one beta rhizobium genotype, most closely related to *Burkholderia graminis*, was isolated.

### Legume–rhizobium interaction networks and nodule occupancy

**Figure 4.**
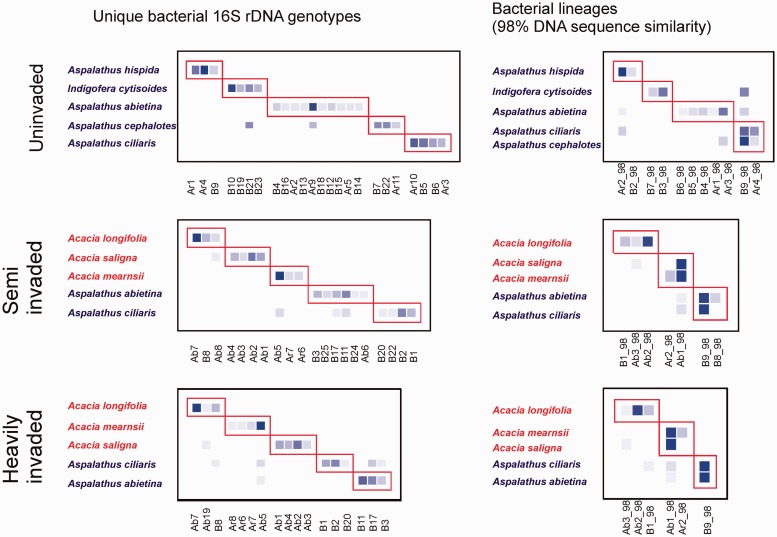
Networks representing legume–rhizobium interactions across a gradient of acacia invasion (uninvaded, semi invaded and heavily invaded sites) for bacterial taxa defined as individual genotypes (see Figs. 1 and 2) and at the 98% 16S rDNA sequence similarity level. Rows represent plant taxa (red species names: invasives, blue species names: natives). Columns represent nodule associated bacterial taxa (B – beta rhizobia in the genus *Burkholderia*, Ar, alpha rhizobia in the *Rhizobium* clade (Fig. 2, clade B); Ab, alpha rhizobia in the *Bradyrhizobium* clade (Fig. 2, clade A)). Increasing intensity of blue represents the increasing frequency of species interactions. Red boxes represent modules identified by the weighted modularity approach of Dormann and Strauss (2014). The invasive and native plant species always occupy distinct modules.

Regardless of how bacterial taxa were delineated (i.e. from genotype to 95% DNA sequence similarity levels) interaction networks at all sites were always significantly modular (results reported for genotype and 98% levels in Table 2 and Figure 4, **[see Supporting Information]** with native and invasive legumes always occupying different modules (Fig. 4, **[see Supporting Information]**). At the bacterial genotype level each of the five legume species at each site formed a module with a unique set of bacteria (Fig. 4), while at the 98% DNA similarity level four modules were recognized at the uninvaded site and three at each of the invaded sites (two modules containing invasive legumes and one containing the natives). At the 95% DNA similarity level networks comprise two modules, one containing the natives and the other the invasives [**see Supporting Information]**. Network topology did not differ markedly across the invasion gradient (Table 2, **[see Supporting Information]**) and species level metrics did not differ significantly between sites **[see Supporting Information]**. All networks were not significantly nested, weakly connected (connectance < 0.55) with an even spread of interactions (IE > 0.57) and high levels of network specialization (H’_2_ > 0.61). Species level metrics did not differ between native and invasive legumes (degree: natives (n) = 4.9 ± 1.5, invasives (i) = 3.8 ± 1.0, F_1,6 _=_ _1.25 ^ns^; effective partners: *n* = 4.1 ± 1.1, *i* = 2.7 ± 1.2, *F*_1,6 _=_ _1.25 ^ns^; specialization: *n* = 0.85 ± 0.10, *i* = 0.95 ± 0.03, *F*_1,6 _=_ _2.49 ^ns^).

**Table 2.** Network level metrics for the uninvaded, semi invaded, heavily invaded and combined interaction networks from analyses (i) treating bacterial genotypes as taxa (*G*) and (ii) identifying bacterial taxa on the basis of 98% 16S sequence similarity (98%). Five plant species were sampled at each site and eight across the combined dataset. Webs were significantly modular with high levels of interaction specialization.

	Bacterial taxa (B)		Connectance (I/P*B)		Interaction evenness (IE)		WNODF		Weighted modularity (Q)		Number of modules		Network specialization (H’_2_)		Generality plant (Gp)		Generality bacteria (Gb)	
	
	G	98%	G	98%	G	98%	G	98%	G	98%	G	98%	G	98%	G	98%	G	98%
Uninvaded	24	11	0.22	0.33	0.63	0.63	5.40^ns^	22.31^ns^	0.72*	0.53*	5	4	0.90	0.68	4.21	2.78	1.19	2.24
Semi invaded	20	7	0.24	0.34	0.61	0.59	8.00^ns^	9.68^ns^	0.74*	0.60*	5	3	0.88	0.89	3.63	1.68	1.20	1.98
Heavily invaded	17	6	0.27	0.40	0.62	0.60	5.88^ns^	12.00^ns^	0.71*	0.59*	5	3	0.85	0.86	3.36	1.55	1.30	2.04
Combined	44	16	0.15	0.23	0.60	0.57	7.92^ns^	15.88^ns^	0.78*	0.59*	7	5	0.89	0.73	5.61	2.31	1.26	2.49

* *P* < 0.05 from *z*-scores. ns, indicates no significant nestedness from randomization tests.

Major clade level composition of nodule occupying rhizobia differed significantly between native and invasive legumes at the invaded sites (*G* = 154.7, df 321 = 2, *P* 0.0001, Table 1) with invasives predominantly associated with alpha rhizobia in the *Bradyrhizobium* clade and natives with beta rhizobia from the genus *Burkholderia* (also see Figs. 1 and 2). Interestingly the composition of rhizobia associated with the native legumes *A. ciliaris* and *A. abietina* differed significantly between uninvaded and invaded sites (*G* = 44.2, df = 2, *P* < 0.0001). In these species alpha rhizobia in the fast-growing ‘*Rhizobium*’ clade (Fig. 1, clade B) were present in more than 50% of nodules at the uninvaded site but were completely absent at the invaded sites (Table 1).

## Discussion

### Structure and changes of legume–rhizobium interaction networks in response to invasion

The legume–rhizobium interaction networks we identified were always strikingly modular and were not nested (Table 2, Fig. 4). This is in contrast with the structure of interaction webs reported from most other mutualisms (e.g. pollination and seed dispersal, Bascompte *et al.* 2003; mycorrhizae, Montesinos-Navarro *et al.* 2012), which tend to be significantly nested, with specialists interacting with a subset of species linked to generalists. Moreover, modules of strongly interacting species weakly linked to other such modules as observed here is perhaps indicative of high levels of evolutionary specialization in this mutualism, something which is confirmed by consistently high estimates of network and species level specialization in our webs (Table 2, Fig. 4). Coevolutionary dynamics, which would drive specialization, are well documented for legume–rhizobium interactions (e.g. Heath and Tiffin, 2009). The architecture of the networks we constructed, the first reported for legume–rhizobium interactions, provide further support for the role of co-evolutionary dynamics in structuring legume–rhizobium interactions.

Some evidence for specialization has been found for CFR legumes. For example, Lemaire *et al.* (2015a, 2016) found that CFR legumes, particularly those in different tribes, associate preferentially with certain alpha and beta rhizobia. Host plants can exert active selection on the rhizobial communities they associate with, even at the genotype level (Heath and Tiffin, 2007). This mechanism likely explains one of the striking patterns in our dataset, i.e. that every plant species sampled at each site associates with a unique set of 16S rRNA bacterial genotypes (i.e. forms a module), this despite multiple plant species associating with the same bacterial taxa as delineated at the 99, 98 and 95% DNA sequence similarity levels. This surprising pattern would arise if populations/lineages within each bacterial taxon evolve in response to selection imposed by different plant taxa, i.e. they represent plant species-specific ecotypes of bacterial lineages (e.g. Heath and Tiffin, 2007). However, Lemaire *et al.* (2015b) have found high phylogenetic incongruence between functional (nodulation) and core genomic gene regions, indicating that HGT is commonplace, in some instances, even between different rhizobial genera. Moreover, host specificity is linked to functional genes and therefore nodulation genes would be the best predictor of co-evolution (Rogel *et al.* 2011). Therefore our results should be treated with caution as it remains to be determined whether distinct 16S rRNA bacterial genotypes represent functionally divergent bacterial strains (i.e. exhibit congruent patterns for nodulation genes) and how the observed patterns will hold up when sampling the same legumes from other habitats.

### Invasive Acacias do not infiltrate native networks, but form novel ‘invasion compartments’

Our findings also suggest, in contrast to what has been shown for other mutualistic networks (Traveset and Richardson, 2014), that invasive acacias do not infiltrate existing native legume–rhizobium interaction networks. Instead they integrate into existing communities as novel modules consisting of interacting legume (acacias) and rhizobia (mainly *Bradyrhizobium* spp.) taxa that are not present in native interaction webs. Although we may have underestimated the diversity of bradyrhizobia based on 16S rDNA diversity alone (e.g. see Vinuesa *et al.* 2008), it is evident that acacias associate with a unique subset of the overall rhizobial diversity that is not found in association with native legumes. It is noteworthy though that we identified some rare and novel associations between *A. mearnsii* and fast growing alpha rhizobia (Fig. 2) and *A.* *longifolia* and beta rhizobia (Fig. 3), indicating that novel associations do occur, albeit infrequently. However, the strength and effectiveness of these associations need to be evaluated and confirmed testing Koch's postulates: re-inoculation with specific rhizobia followed by successful re-isolation from newly formed nodules. Overall our results suggest that invasive acacia-rhizobium associations reflect a lack of overlap with native legume symbiont requirements. That is, native CFR legumes do not appear to associate with *Bradyrhizobium* strains, even when co-occurring with acacias. This is also true for natural populations of CFR legumes in general (Lemaire *et al.* 2015a). Similar to our study, Weir *et al.* (2004) found acacias in New Zealand to be exclusively nodulated by *Bradyrhizobium* strains, while native legumes were mostly nodulated by *Mesorhizobium* strains. Liu *et al.* (2014) also found invasive South African *Dipogon lignosus* in New Zealand to be nodulated by strains of South African-type *Burkholderia*, and that these do not appear to nodulate any native New Zealand legumes.

The phylogenetic distinctiveness of native legumes (four of five species from the genus *Aspalathus*) and invasive acacias may also explain the apparent lack of mutualist overlap observed between these two groups. Symbiotic preferences and effectiveness often show host plant phylogenetic signal (e.g. Dobert *et al.* 1994), and it is expected that more closely related legumes perform better when sharing rhizobia compared with distantly related species. However, together with the low prevalence of *Bradyrhizobium* in CFR soils (Lemaire *et al.* 2015a), our data support the possibility that acacias have been co-introduced to the CFR with suitable mutualistic bacteria, a phenomenon that has been observed for some woody invaders, including acacias (Nuñez and Dickie 2014). It is thought that rhizobia are either directly co-introduced with their host plants as inoculants for agroforestry (Marques *et al.* 2001) or indirectly as hitchhikers on introduced plant material (Porter *et al.* 2011; Weir *et al.* 2004. Co-invasions of acacias and their associated alpha rhizobia have been demonstrated in South Africa for *A. pycnantha* (Ndlovu *et al.* 2013) and for *A. longifolia* and *A. saligna* in Europe (Crisóstomo *et al.* 2013; Rodríguez-Echeverría 2010). The co-introduction of legumes and beta rhizobia is also commonplace. For example, various studies have demonstrated independent co-introductions of *Mimosa* species and their associated beta rhizobia from their native Neotropics ranges to Australia (Parker *et al.* 2007), China (Liu *et al.* 2012) and Taiwan (Chen *et al.* 2005). For this group of legumes the potential importance of co-introductions for establishment success is exemplified by *M. pudica* invasions in India. Here, invasive populations only nodulated with co-introduced beta rhizobia and appear unable to utilize alpha rhizobia associated with co-occurring Indian *Mimosa* species (Gehlot *et al.* 2013). It is therefore easy to recognize how co-introductions can lead to non-overlapping symbiont requirements with native legumes, high modularity and co-evolved specialization, as observed in our study.

Our 16S rRNA sequencing results did not allow us to eliminate the possibility that free-living *Bradyrhizobium* strains, with which invasive acacias nodulate, are natural components, albeit in very low densities, of the CFR. What we do know is that *Bradyrhizobium* is almost never, or extremely infrequently, associated with most CFR legumes investigated to date (e.g. Kock 2004; Lemaire *et al.* 2015a, Fig. 4). This, together with the discreet native and invasive modules identified in our networks, suggest that acacias are not associating with native rhizobia, but are likely forming associations with bacteria which are themselves invasive.

Another surprising finding was that invasive acacias, despite all predominantly interacting with bradyrhizobia, do not occupy a single interaction module (at all but the 95% DNA sequence similarity level [**see** **Supporting Information** Fig. 2]). Specifically, *A.* *longifolia* interacts with a different *Bradyrhizobium* strain than *A. saligna* and *A. mearnsii* (Fig. 2). Again, these unique associations are based on identity alone and functional genes, e.g. *nodD*, may collapse acacias into a single interaction module because of functional similarity between different acacia-associated rhizobia. Interestingly, Crisóstomo *et al.* (2013) recently reported that *A. longifolia* and *A. saligna* also nodulate with qualitatively different bacterial communities in their native Australian range. However, in some instances it has been found that different Australian acacias share some of their rhizobial symbionts, both in their native (e.g. Thrall *et al.* 2007) and introduced ranges (e.g. Rodríguez-Echeverría *et al.* 2011). Future research to disentangle the sources of invader-associated rhizobia should be directed towards incorporating multiple genes for rhizobial identification, including functional genes, and native range provenances.

### Native nodule rhizobium communities exhibit near-complete compositional turnover across the invasion gradi*e*nt

No native legumes were found to nodulate with bradyrhizobia in uninvaded soils, while these constituted 86% of acacia-associated symbionts. At invaded sites 8% of native nodules were occupied by bradyrhizobia (i.e. constitute novel interactions). Thus, despite the apparent change towards bradyrhizobia-dominated microbial communities across the invasion gradient, native legumes appear not to associate with these rhizobia, perhaps either as a result of co-evolved specialization to their associated rhizobia or perhaps through discrimination against possibly symbiotically less efficient bradyrhizobia. Similarly, Lemaire *et al.* (2015a) reported that very few native CFR legumes, including species from the two genera included in our study, nodulate with *Bradyrhizobium* strains, and that most CFR legumes associate with *Burkholderia* and *Mesorhizobium* strains. Tolerance to acidic soil conditions by both legumes and rhizobia is a pre-requisite for effective nodulation in the CFR. For example, common CFR legume associates in the genera *Burkholderia* and *Mesorhizobium* are adapted to acidic soils (Garau *et al.* 2009; Lemaire *et al.* 2015a). One has to assume that the same holds true for acacia-associated bradyrhizobia. Indeed, the bradyrhizobia associated with some indigenous South African legumes appear highly tolerant of extremely low soil pH conditions (Muofhe and Dakora 1997).

The composition of native symbiont communities did however change dramatically across the invasion gradient. The composition of rhizobia associated with the two native legumes present at all sites, *A.* *ciliaris* and *A. abietina*, differed significantly between uninvaded and invaded sites (*G* = 44.2, df = 2, *P* < 0.0001). In these species alpha rhizobia in the fast-growing ‘*Rhizobium’* clade (Fig. 1, clade B) were present in more than 50% of nodules at the uninvaded site but were completely absent at the invaded sites (Table 2). This raises the intriguing possibility that bacterial composition change involves competitive displacement of other alpha bacteria by acacia-associated *Bradyrhizobium* spp., something that would certainly be worth exploring experimentally or by sampling across replicate invasion gradients. Native legume species that persisted under invasion also appear to be more generalist in their bacterial interactions, in the sense that they exploited beta and alpha rhizobia with equal probability at the uninvaded site, whilst plant species which were absent under invasion tended to specialize on one or the other of these bacterial clades (Table 2). Without replication and proper experimental approaches it is not possible for us to distinguish whether symbiont limitations are driving the exclusion of native legumes in heavily invaded sites or whether other intrinsic/extrinsic factors (e.g. soil chemistry, competition, etc.) are the main drivers of this pattern.

## Conclusions

Our findings indicate that, like many other plant-mutualism interactions, native legume–rhizobium interactions are impacted by the presence of invasive species in communities. However, for invasive Australian acacias these impacts contradict conventional wisdom, with acacias being specialized and not infiltrating existing native networks. Instead, acacias and their symbionts form novel modules which are largely unconnected to highly modular native legume–rhizobium networks. Our results illustrate a clear need to better understand the influence of positive feedbacks in altering belowground community level interactions during invasion and how these may impact and limit efforts to restore invaded systems. Moreover, contrasting the network structures obtained here against those observed for functional genes involved in the legume–rhizobium mutualism process, e.g. nodulation (e.g. *NodA*, *B* or *C*), may prove particularly interesting. Legume–rhizobium specialization mediated by nodulation genes might reduce the number of detected modules, relative to 16S rDNA, for closely related species (e.g. acacias) due to HGT of similar functional symbiosis genes. Even so, we predict that the strong differentiation between native legumes and acacias is likely to remain due to distinct host plant phylogenetic histories and the lack of HGT between major rhizobial lineages (e.g. alpha vs. beta rhizobia). Our results provide a promising first step in understanding how legume–rhizobium networks are structured and how they react to biological invasions. Incorporating the approaches used here across mulitiple sites and/or using different legume taxa (native and invasive) opens the door for interesting, much needed and promising future research opportunities.

## Accession Numbers

KU836936–KU837225

## Sources of Funding

Funding for this research was provided by the DST-NRF Centre of Excellence for Invasion Biology (C•I•B), Stellenbosch University (through the office of the Vice Rector: Research, Innovation and Postgraduate Studies), and the South African National Research Foundation (NRF, DVGR grant no. 98182 awarded to J.L.R.). We also acknowledge the financial support from the C•I•B and the Working for Water Programme through the collaborative research project on ‘Research for Integrated Management of Invasive Alien Species’. J.L.R. also acknowledges support from the Oppenheimer Memorial Trust (grant 19820/02), the HB & MJ Thom Award (Stellenbosch University) for sabbatical research funding, the South African National Research Foundation (NRF, grant no. 93591), National Geographic, Committee for Research and Exploration grant (grant no. 9250-13), and British Ecological Society 'Ecologists in Africa' grant (grant no. 4101-4991).

## Contributions by the Authors

J.L.R. and A.G.E. contributed equally in the conceptualisation of the research, analyses of the data, and in leading the writing of the manuscript. N.M. generated genetic data and contributed to the writing of the manuscript.

## Conflicts of Interest Statement

None declared.

## Supplementary Material

Supplementary Data

## Supporting Information

The following additional information is available in the online version of this article —

**File 1.** Table. Lists the host, locality and DNA sequence information for rhizobial isolations included in this study.

**File 2.** Table. Provides network metrics for all bacterial taxa delineation (based on DNA similarity) levels.

**File 3.** Table. Lists species level network metrics for plants and bacteria at the genotype and 98% similarity levels.

**File 4.** Illustrates interaction networks of all legume-rhizobia interactions across a gradient of *Acacia* invasion (uninvaded, semi invaded and heavily invaded) as well as for the combined dataset, for hierarchically delineated rhizobial taxa (genotype, 99%, 98% and 95% DNA sequence similarity levels).
