# Supplementary material for: The structure of legume–rhizobium interaction networks and their response to tree invasions
Source: AoB Plants. 2016 Jul 11;8:plw038. doi: 10.1093/aobpla/plw038 (PMC4940501; doi:10.1093/aobpla/plw038)
Supplement: Supplementary Data [file supp_8_plw038_index.html]

The structure of legume–rhizobium interaction networks and their response to tree invasions — Supplementary Data 

# The structure of legume–rhizobium interaction networks and their response to tree invasions

## Supplementary Data

files

- Supplementary Data - docx file
