# Supplementary material for: The structure of legume–rhizobium interaction networks and their response to tree invasions
Source: AoB Plants. 2016 Jul 11;8:plw038. doi: 10.1093/aobpla/plw038 (PMC4940501; doi:10.1093/aobpla/plw038)
Supplement: Supplementary Data [file supp_plw038_aobplants-16008-s02.docx]

**File 1**: Host, locality and DNA sequence information for rhizobial isolations included in this study.

| **Betaproteobacteria** |  |  |  |
| --- | --- | --- | --- |
| **Isolate number** | **Host plant** | **Site** | **GenBank Accession number** |
| VG1_1a_pre | *Aspalathus abietina* | uninvaded | KU837006 |
| VG1_1b_pre | *Aspalathus abietina* | uninvaded | KU837007 |
| VG1_2C_pre | *Aspalathus abietina* | uninvaded | KU836957 |
| VG1_3C_pre | *Aspalathus abietina* | uninvaded | KU837060 |
| VG1_3d_pre | *Aspalathus abietina* | uninvaded | KU837061 |
| VG1_4B_pre | *Aspalathus abietina* | uninvaded | KU837062 |
| VG1_4C_pre | *Aspalathus abietina* | uninvaded | KU837063 |
| VG1_5B_pre | *Aspalathus abietina* | uninvaded | KU837064 |
| VG1_5C_pre | *Aspalathus abietina* | uninvaded | KU837065 |
| VG1_1A_sem | *Aspalathus abietina* | semi invaded | KU836979 |
| VG1_1d_sem | *Aspalathus abietina* | semi invaded | KU836978 |
| VG1_1b_sem | *Aspalathus abietina* | semi invaded | KU836994 |
| VG1_1c_sem | *Aspalathus abietina* | semi invaded | KU837065 |
| VG1_2a_sem | *Aspalathus abietina* | semi invaded | KU836959 |
| VG1_2b_sem | *Aspalathus abietina* | semi invaded | KU836960 |
| VG1_2C_sem | *Aspalathus abietina* | semi invaded | KU836975 |
| VG1_2d_sem | *Aspalathus abietina* | semi invaded | KU836962 |
| VG1_3A_sem | *Aspalathus abietina* | semi invaded | KU836976 |
| VG1_3b_sem | *Aspalathus abietina* | semi invaded | KU836974 |
| VG1_3C_sem | *Aspalathus abietina* | semi invaded | KU836958 |
| VG1_3c_sem | *Aspalathus abietina* | semi invaded | KU836958 |
| VG1_4a_sem | *Aspalathus abietina* | semi invaded | KU836980 |
| VG1_4b_sem | *Aspalathus abietina* | semi invaded | KU836993 |
| VG1_4C1_sem | *Aspalathus abietina* | semi invaded | KU836997 |
| VG1_4C2_sem | *Aspalathus abietina* | semi invaded | KU836995 |
| VG1_5a_sem | *Aspalathus abietina* | semi invaded | KU836961 |
| VG1_5C_sem | *Aspalathus abietina* | semi invaded | KU836977 |
| VG1_1A_inv | *Aspalathus abietina* | heavily invaded | KU836990 |
| VG1_1b_inv | *Aspalathus abietina* | heavily invaded | KU836968 |
| VG1_1c_inv | *Aspalathus abietina* | heavily invaded | KU836967 |
| VG1_1d_inv | *Aspalathus abietina* | heavily invaded | KU836989 |
| VG1_2a_inv | *Aspalathus abietina* | heavily invaded | KU836969 |
| VG1_2b_inv | *Aspalathus abietina* | heavily invaded | KU836970 |
| VG1_2C_inv | *Aspalathus abietina* | heavily invaded | KU836987 |
| VG1_2d_inv | *Aspalathus abietina* | heavily invaded | KU836973 |
| VG1_3A_inv | *Aspalathus abietina* | heavily invaded | KU836983 |
| VG1_3b_inv | *Aspalathus abietina* | heavily invaded | KU836984 |
| VG1_3C_inv | *Aspalathus abietina* | heavily invaded | KU836971 |
| VG1_3d_inv | *Aspalathus abietina* | heavily invaded | KU836988 |
| VG1_4a_inv | *Aspalathus abietina* | heavily invaded | KU836991 |
| VG1_4b_inv | *Aspalathus abietina* | heavily invaded | KU836998 |
| VG1_4C1_inv | *Aspalathus abietina* | heavily invaded | KU836992 |
| VG1_4C2_inv | *Aspalathus abietina* | heavily invaded | KU836999 |
| VG1_5a_inv | *Aspalathus abietina* | heavily invaded | KU836972 |
| VG1_5C_inv | *Aspalathus abietina* | heavily invaded | KU836985 |
| VG1_5d_inv | *Aspalathus abietina* | heavily invaded | KU836986 |
| VG2_3a_pre | *Aspalathus hispida* | uninvaded | KU837040 |
| VG2_3b_pre | *Aspalathus hispida* | uninvaded | KU837041 |
| VG3_1a_pre | *Aspalathus ciliaris* | uninvaded | KU836981 |
| VG3_1B_pre | *Aspalathus ciliaris* | uninvaded | KU837000 |
| VG3_1C_pre | *Aspalathus ciliaris* | uninvaded | KU837001 |
| VG3_2B_pre | *Aspalathus ciliaris* | uninvaded | KU837032 |
| VG3_2D_pre | *Aspalathus ciliaris* | uninvaded | KU837002 |
| VG3_3A_pre | *Aspalathus ciliaris* | uninvaded | KU837034 |
| VG3_3D_pre | *Aspalathus ciliaris* | uninvaded | KU837033 |
| VG3_4A_pre | *Aspalathus ciliaris* | uninvaded | KU837004 |
| VG3_4B_pre | *Aspalathus ciliaris* | uninvaded | KU837003 |
| VG3_5A_pre | *Aspalathus ciliaris* | uninvaded | KU837005 |
| VG3_1a_sem | *Aspalathus ciliaris* | semi invaded | KU837031 |
| VG3_1b_sem | *Aspalathus ciliaris* | semi invaded | KU836939 |
| VG3_2a_sem | *Aspalathus ciliaris* | semi invaded | KU836955 |
| VG3_2b_sem | *Aspalathus ciliaris* | semi invaded | KU837014 |
| VG3_2C_sem | *Aspalathus ciliaris* | semi invaded | KU837012 |
| VG3_2D_sem | *Aspalathus ciliaris* | semi invaded | KU837013 |
| VG3_3a_sem | *Aspalathus ciliaris* | semi invaded | KU837008 |
| VG3_3B_sem | *Aspalathus ciliaris* | semi invaded | KU837009 |
| VG3_3C_sem | *Aspalathus ciliaris* | semi invaded | KU836963 |
| VG3_3D_sem | *Aspalathus ciliaris* | semi invaded | KU837010 |
| VG3_4A_sem | *Aspalathus ciliaris* | semi invaded | KU837011 |
| VG3_4b_sem | *Aspalathus ciliaris* | semi invaded | KU837022 |
| VG3_4c_sem | *Aspalathus ciliaris* | semi invaded | KU837023 |
| VG3_4D_sem | *Aspalathus ciliaris* | semi invaded | KU837024 |
| VG3_5a_sem | *Aspalathus ciliaris* | semi invaded | KU836982 |
| VG3_5b_sem | *Aspalathus ciliaris* | semi invaded | KU837025 |
| VG3_1a_inv | *Aspalathus ciliaris* | heavily invaded | KU836956 |
| VG3_1b_inv | *Aspalathus ciliaris* | heavily invaded | KU837042 |
| VG3_1c_inv | *Aspalathus ciliaris* | heavily invaded | KU836996 |
| VG3_1d_inv | *Aspalathus ciliaris* | heavily invaded | KU836964 |
| VG3_2a_inv | *Aspalathus ciliaris* | heavily invaded | KU836965 |
| VG3_2b_inv | *Aspalathus ciliaris* | heavily invaded | KU837015 |
| VG3_2C_inv | *Aspalathus ciliaris* | heavily invaded | KU837016 |
| VG3_2D_inv | *Aspalathus ciliaris* | heavily invaded | KU837017 |
| VG3_3a_inv | *Aspalathus ciliaris* | heavily invaded | KU837018 |
| VG3_3B_inv | *Aspalathus ciliaris* | heavily invaded | KU837019 |
| VG3_3C_inv | *Aspalathus ciliaris* | heavily invaded | KU836966 |
| VG3_3D_inv | *Aspalathus ciliaris* | heavily invaded | KU837020 |
| VG3_4A_inv | *Aspalathus ciliaris* | heavily invaded | KU837021 |
| VG3_4b_inv | *Aspalathus ciliaris* | heavily invaded | KU837026 |
| VG3_4c_inv | *Aspalathus ciliaris* | heavily invaded | KU837027 |
| VG3_4D_inv | *Aspalathus ciliaris* | heavily invaded | KU837028 |
| VG3_5a_inv | *Aspalathus ciliaris* | heavily invaded | KU837029 |
| VG3_5b_inv | *Aspalathus ciliaris* | heavily invaded | KU837030 |
| VG4_1c_pre | *Aspalathus cephalotes* subsp. *violacea* | uninvaded | KU837035 |
| VG4_1d_pre | *Aspalathus cephalotes* subsp. *violacea* | uninvaded | KU837036 |
| VG4_2a_pre | *Aspalathus cephalotes* subsp. *violacea* | uninvaded | KU837037 |
| VG4_2b_pre | *Aspalathus cephalotes* subsp. *violacea* | uninvaded | KU837038 |
| VG4_2d_pre | *Aspalathus cephalotes* subsp. *violacea* | uninvaded | KU837039 |
| VG4_3a_pre | *Aspalathus cephalotes* subsp. *violacea* | uninvaded | KU836940 |
| VG4_3b_pre | *Aspalathus cephalotes* subsp. *violacea* | uninvaded | KU836941 |
| VG4_3c_pre | *Aspalathus cephalotes* subsp. *violacea* | uninvaded | KU836942 |
| VG4_3d_pre | *Aspalathus cephalotes* subsp. *violacea* | uninvaded | KU836943 |
| VG4_4a_pre | *Aspalathus cephalotes* subsp. *violacea* | uninvaded | KU836944 |
| VG4_4b_pre | *Aspalathus cephalotes* subsp. *violacea* | uninvaded | KU836950 |
| VG4_4C_pre | *Aspalathus cephalotes* subsp. *violacea* | uninvaded | KU836951 |
| VG4_5b_pre | *Aspalathus cephalotes* subsp. *violacea* | uninvaded | KU836952 |
| VG4_5c_pre | *Aspalathus cephalotes* subsp. *violacea* | uninvaded | KU836953 |
| VG4_5d_pre | *Aspalathus cephalotes* subsp. *violacea* | uninvaded | KU836954 |
| Ind_1c_pre | *Indigofera cytisoides* | uninvaded | KU837051 |
| Ind_1d_pre | *Indigofera cytisoides* | uninvaded | KU837052 |
| Ind_2a_pre | *Indigofera cytisoides* | uninvaded | KU837053 |
| Ind_2b_pre | *Indigofera cytisoides* | uninvaded | KU837054 |
| Ind_2c_pre | *Indigofera cytisoides* | uninvaded | KU836936 |
| Ind_2d_pre | *Indigofera cytisoides* | uninvaded | KU836937 |
| Ind_3a_pre | *Indigofera cytisoides* | uninvaded | KU837055 |
| Ind_3b_pre | *Indigofera cytisoides* | uninvaded | KU836938 |
| Ind_3c_pre | *Indigofera cytisoides* | uninvaded | KU836945 |
| Ind_3d_pre | *Indigofera cytisoides* | uninvaded | KU836946 |
| Ind_4b_pre | *Indigofera cytisoides* | uninvaded | KU837056 |
| Ind_4c_pre | *Indigofera cytisoides* | uninvaded | KU836947 |
| Ind_4d_pre | *Indigofera cytisoides* | uninvaded | KU836948 |
| Ind_5a_pre | *Indigofera cytisoides* | uninvaded | KU837057 |
| Ind_5b_pre | *Indigofera cytisoides* | uninvaded | KU837058 |
| Ind_5c_pre | *Indigofera cytisoides* | uninvaded | KU836949 |
| Ind_5d_pre | *Indigofera cytisoides* | uninvaded | KU837059 |
| AL_2a_sem | *Acacia longifolia* | semi invaded | KU837043 |
| AL_3a_sem | *Acacia longifolia* | semi invaded | KU837049 |
| AL_3D_sem | *Acacia longifolia* | semi invaded | KU837044 |
| AL_5C_sem | *Acacia longifolia* | semi invaded | KU837045 |
| AL_2D_inv | *Acacia longifolia* | heavily invaded | KU837046 |
| AL_3B_inv | *Acacia longifolia* | heavily invaded | KU837050 |
| AL_3D_inv | *Acacia longifolia* | heavily invaded | KU837047 |
| AL_4C_inv | *Acacia longifolia* | heavily invaded | KU837048 |
|  | | | |
| **Alphaproteobacteria** | | | |
| **Isolate number** | **Host plant** | **Site** | **GenBank** |
| VG1_1c_pre | *Aspalathus abietina* | uninvaded | KU837214 |
| VG1_1d_pre | *Aspalathus abietina* | uninvaded | KU837215 |
| VG1_2a_pre | *Aspalathus abietina* | uninvaded | KU837216 |
| VG1_2b_pre | *Aspalathus abietina* | uninvaded | KU837213 |
| VG1_2D_pre | *Aspalathus abietina* | uninvaded | KU837103 |
| VG1 3a_pre | *Aspalathus abietina* | uninvaded | KU837217 |
| VG1_3b_pre | *Aspalathus abietina* | uninvaded | KU837218 |
| VG1_4a_pre | *Aspalathus abietina* | uninvaded | KU837219 |
| VG1_4D_pre | *Aspalathus abietina* | uninvaded | KU837220 |
| VG1_5a_pre | *Aspalathus abietina* | uninvaded | KU837221 |
| VG1_5d_pre | *Aspalathus abietina* | uninvaded | KU837222 |
| VG1 5B sem | *Aspalathus abietina* | semi invaded | KU837212 |
| VG1_5b_inv | *Aspalathus abietina* | heavily invaded | KU837136 |
| VG2_1a_pre | *Aspalathus hispida* | uninvaded | KU837077 |
| VG2_1b_pre | *Aspalathus hispida* | uninvaded | KU837078 |
| VG2_1c_pre | *Aspalathus hispida* | uninvaded | KU837079 |
| VG2_1D_pre | *Aspalathus hispida* | uninvaded | KU837080 |
| VG2_2a_pre | *Aspalathus hispida* | uninvaded | KU837081 |
| VG2_2C_pre | *Aspalathus hispida* | uninvaded | KU837085 |
| VG2_2D_pre | *Aspalathus hispida* | uninvaded | KU837086 |
| VG2_3c_pre | *Aspalathus hispida* | uninvaded | KU837087 |
| VG2_3D_pre | *Aspalathus hispida* | uninvaded | KU837088 |
| VG2_4A_pre | *Aspalathus hispida* | uninvaded | KU837089 |
| VG2_4B_pre | *Aspalathus hispida* | uninvaded | KU837090 |
| VG2_4C_pre | *Aspalathus hispida* | uninvaded | KU837091 |
| VG2_4D_pre | *Aspalathus hispida* | uninvaded | KU837082 |
| VG2_5A_pre | *Aspalathus hispida* | uninvaded | KU837083 |
| VG2_5B_pre | *Aspalathus hispida* | uninvaded | KU837084 |
| VG2_5C_pre | *Aspalathus hispida* | uninvaded | KU837093 |
| VG2_5D_pre | *Aspalathus hispida* | uninvaded | KU837094 |
| VG3_1d_pre | *Aspalathus ciliaris* | uninvaded | KU837066 |
| VG3_2a_pre | *Aspalathus ciliaris* | uninvaded | KU837067 |
| VG3_3B_pre | *Aspalathus ciliaris* | uninvaded | KU837068 |
| VG3_3c_pre | *Aspalathus ciliaris* | uninvaded | KU837074 |
| VG3_4C_pre | *Aspalathus ciliaris* | uninvaded | KU837069 |
| VG3_4D_pre | *Aspalathus ciliaris* | uninvaded | KU837075 |
| VG3_5B_pre | *Aspalathus ciliaris* | uninvaded | KU837070 |
| VG3_5C_pre | *Aspalathus ciliaris* | uninvaded | KU837076 |
| VG3_5D_pre | *Aspalathus ciliaris* | uninvaded | KU837071 |
| VG3 1c sem | *Aspalathus ciliaris* | semi invaded | KU837137 |
| VG3 1d sem | *Aspalathus ciliaris* | semi invaded | KU837138 |
| VG3 5C inv | *Aspalathus ciliaris* | heavily invaded | KU837177 |
| VG3 5d inv | *Aspalathus ciliaris* | heavily invaded | KU837178 |
| VG4_1a_pre | *Aspalathus cephalotes* subsp. *violacea* | uninvaded | KU837072 |
| VG4_1b_pre | *Aspalathus cephalotes* subsp. *violacea* | uninvaded | KU837223 |
| VG4_2c_pre | *Aspalathus cephalotes* subsp. *violacea* | uninvaded | KU837073 |
| VG4_4D_pre | *Aspalathus cephalotes* subsp. *violacea* | uninvaded | KU837224 |
| VG4_5a_pre | *Aspalathus cephalotes* subsp. *violacea* | uninvaded | KU837225 |
| AL_1A_sem | *Acacia longifolia* | semi invaded | KU837179 |
| AL_1B_sem | *Acacia longifolia* | semi invaded | KU837180 |
| AL_1C_sem | *Acacia longifolia* | semi invaded | KU837181 |
| AL_1d_sem | *Acacia longifolia* | semi invaded | KU837182 |
| AL_2A_sem | *Acacia longifolia* | semi invaded | KU837183 |
| AL_2B_sem | *Acacia longifolia* | semi invaded | KU837184 |
| AL_2C_sem | *Acacia longifolia* | semi invaded | KU837195 |
| AL_3A_sem | *Acacia longifolia* | semi invaded | KU837204 |
| AL_3C_sem | *Acacia longifolia* | semi invaded | KU837203 |
| AL_4a_sem | *Acacia longifolia* | semi invaded | KU837189 |
| AL_4B_sem | *Acacia longifolia* | semi invaded | KU837196 |
| AL_4D_sem | *Acacia longifolia* | semi invaded | KU837207 |
| AL_5A_sem | *Acacia longifolia* | semi invaded | KU837197 |
| AL_5B_sem | *Acacia longifolia* | semi invaded | KU837190 |
| AL_5C_sem | *Acacia longifolia* | semi invaded | KU837191 |
| AL_5D_sem | *Acacia longifolia* | semi invaded | KU837208 |
| AL_1A_inv | *Acacia longifolia* | heavily invaded | KU837185 |
| AL_1C_inv | *Acacia longifolia* | heavily invaded | KU837186 |
| AL_1d_inv | *Acacia longifolia* | heavily invaded | KU837187 |
| AL_2A_inv | *Acacia longifolia* | heavily invaded | KU837188 |
| AL_2B_inv | *Acacia longifolia* | heavily invaded | KU837198 |
| AL_2C_inv | *Acacia longifolia* | heavily invaded | KU837199 |
| AL_3A_inv | *Acacia longifolia* | heavily invaded | KU837206 |
| AL_3C_inv | *Acacia longifolia* | heavily invaded | KU837205 |
| AL_4a_inv | *Acacia longifolia* | heavily invaded | KU837200 |
| AL_4B_inv | *Acacia longifolia* | heavily invaded | KU837201 |
| AL_4D_inv | *Acacia longifolia* | heavily invaded | KU837210 |
| AL_5A_inv | *Acacia longifolia* | heavily invaded | KU837211 |
| AL_5B_inv | *Acacia longifolia* | heavily invaded | KU837192 |
| AL_5C_inv | *Acacia longifolia* | heavily invaded | KU837193 |
| AL_5D_inv | *Acacia longifolia* | heavily invaded | KU837194 |
| AM_1a_sem | *Acacia mearnsii* | semi invaded | KU837104 |
| AM_1b_sem | *Acacia mearnsii* | semi invaded | KU837106 |
| AM_1C_sem | *Acacia mearnsii* | semi invaded | KU837107 |
| AM_1d_sem | *Acacia mearnsii* | semi invaded | KU837108 |
| AM_2A_sem | *Acacia mearnsii* | semi invaded | KU837112 |
| AM_2B_sem | *Acacia mearnsii* | semi invaded | KU837095 |
| AM_2C_sem | *Acacia mearnsii* | semi invaded | KU837134 |
| AM_2D_sem | *Acacia mearnsii* | semi invaded | KU837113 |
| AM_3A_sem | *Acacia mearnsii* | semi invaded | KU837114 |
| AM_3B_sem | *Acacia mearnsii* | semi invaded | KU837115 |
| AM_3C_sem | *Acacia mearnsii* | semi invaded | KU837116 |
| AM_3D_sem | *Acacia mearnsii* | semi invaded | KU837117 |
| AM_4A_sem | *Acacia mearnsii* | semi invaded | KU837121 |
| AM_4B_sem | *Acacia mearnsii* | semi invaded | KU837122 |
| AM_4C_sem | *Acacia mearnsii* | semi invaded | KU837100 |
| AM_4d_sem | *Acacia mearnsii* | semi invaded | KU837096 |
| AM_5A_sem | *Acacia mearnsii* | semi invaded | KU837118 |
| AM_5b_sem | *Acacia mearnsii* | semi invaded | KU837119 |
| AM_5C_sem | *Acacia mearnsii* | semi invaded | KU837120 |
| AM_5D_sem | *Acacia mearnsii* | semi invaded | KU837099 |
| AM_1a_inv | *Acacia mearnsii* | heavily invaded | KU837105 |
| AM_1b_inv | *Acacia mearnsii* | heavily invaded | KU837109 |
| AM_1C_inv | *Acacia mearnsii* | heavily invaded | KU837110 |
| AM_1d_inv | *Acacia mearnsii* | heavily invaded | KU837111 |
| AM_2A_inv | *Acacia mearnsii* | heavily invaded | KU837123 |
| AM_2B_inv | *Acacia mearnsii* | heavily invaded | KU837124 |
| AM_2C_inv | *Acacia mearnsii* | heavily invaded | KU837125 |
| AM_2D_inv | *Acacia mearnsii* | heavily invaded | KU837126 |
| AM_3A_inv | *Acacia mearnsii* | heavily invaded | KU837127 |
| AM_3B_inv | *Acacia mearnsii* | heavily invaded | KU837128 |
| AM_3C_inv | *Acacia mearnsii* | heavily invaded | KU837170 |
| AM_4A_inv | *Acacia mearnsii* | heavily invaded | KU837132 |
| AM_4B_inv | *Acacia mearnsii* | heavily invaded | KU837133 |
| AM_4C_inv | *Acacia mearnsii* | heavily invaded | KU837102 |
| AM_4d_inv | *Acacia mearnsii* | heavily invaded | KU837098 |
| AM_5A_inv | *Acacia mearnsii* | heavily invaded | KU837129 |
| AM_5b_inv | *Acacia mearnsii* | heavily invaded | KU837130 |
| AM_5C_inv | *Acacia mearnsii* | heavily invaded | KU837131 |
| AM_5D_inv | *Acacia mearnsii* | heavily invaded | KU837101 |
| AS_1a_sem | *Acacia saligna* | semi invaded | KU837140 |
| AS_1b_sem | *Acacia saligna* | semi invaded | KU837151 |
| AS_1c_sem | *Acacia saligna* | semi invaded | KU837167 |
| AS_1d_sem | *Acacia saligna* | semi invaded | KU837155 |
| AS_2a_sem | *Acacia saligna* | semi invaded | KU837148 |
| AS_2b_sem | *Acacia saligna* | semi invaded | KU837175 |
| AS_2c_sem | *Acacia saligna* | semi invaded | KU837176 |
| AS_2d_sem | *Acacia saligna* | semi invaded | KU837163 |
| AS_3a_sem | *Acacia saligna* | semi invaded | KU837153 |
| AS_3b_sem | *Acacia saligna* | semi invaded | KU837156 |
| AS_3c_sem | *Acacia saligna* | semi invaded | KU837169 |
| AS_3D_sem | *Acacia saligna* | semi invaded | KU837202 |
| AS_4a_sem | *Acacia saligna* | semi invaded | KU837141 |
| AS_4b_sem | *Acacia saligna* | semi invaded | KU837142 |
| AS_4C_sem | *Acacia saligna* | semi invaded | KU837157 |
| AS_4d_sem | *Acacia saligna* | semi invaded | KU837158 |
| AS_5a_sem | *Acacia saligna* | semi invaded | KU837209 |
| AS_5b_sem | *Acacia saligna* | semi invaded | KU837139 |
| AS_5c_sem | *Acacia saligna* | semi invaded | KU837147 |
| AS_5d_sem | *Acacia saligna* | semi invaded | KU837165 |
| AS_1a_inv | *Acacia saligna* | heavily invaded | KU837144 |
| AS_1b_inv | *Acacia saligna* | heavily invaded | KU837152 |
| AS_1c_inv | *Acacia saligna* | heavily invaded | KU837168 |
| AS_1d_inv | *Acacia saligna* | heavily invaded | KU837159 |
| AS_2a_inv | *Acacia saligna* | heavily invaded | KU837150 |
| AS_2d_inv | *Acacia saligna* | heavily invaded | KU837164 |
| AS_3a_inv | *Acacia saligna* | heavily invaded | KU837154 |
| AS_3b_inv | *Acacia saligna* | heavily invaded | KU837160 |
| AS_3c_inv | *Acacia saligna* | heavily invaded | KU837173 |
| AS_3D_inv | *Acacia saligna* | heavily invaded | KU837174 |
| AS_4a_inv | *Acacia saligna* | heavily invaded | KU837145 |
| AS_4b_inv | *Acacia saligna* | heavily invaded | KU837146 |
| AS_4C_inv | *Acacia saligna* | heavily invaded | KU837161 |
| AS_4d_inv | *Acacia saligna* | heavily invaded | KU837162 |
| AS_5a_inv | *Acacia saligna* | heavily invaded | KU837206 |
| AS_5b_inv | *Acacia saligna* | heavily invaded | KU837143 |
| AS_5c_inv | *Acacia saligna* | heavily invaded | KU837149 |
| AS_5d_inv | *Acacia saligna* | heavily invaded | KU837166 |

**File 2**: Network metrics for all bacterial taxa delineation levels.

|  | Number of bacterial taxa (B) | Connectance (I/P*B) | Interaction evenness (IE) | Weighted nestedness (WNODF) | Weighted modularity (Q) | Number of Modules | Network specialization (H’_2_) | Generality plant (Gp) | Generality bacteria (Gb) |
| --- | --- | --- | --- | --- | --- | --- | --- | --- | --- |
| **Genotype level** | | | | | | | | | |
| Uninvaded | 24 | 0.22 | 0.63 | 5.40 ^ns^ | 0.72* | 5 | 0.90 | 4.21 | 1.19 |
| Semi-invaded | 20 | 0.24 | 0.61 | 8.00 ^ns^ | 0.74* | 5 | 0.88 | 3.63 | 1.20 |
| Invaded | 17 | 0.27 | 0.62 | 5.88 ^ns^ | 0.71* | 5 | 0.85 | 3.36 | 1.30 |
| COMBINED | 44 | 0.15 | 0.60 | 7.92 ^ns^ | 0.78* | 7 | 0.89 | 5.61 | 1.26 |
| **99 % DNA similarity level** | | | | | | | | | |
| Uninvaded | 13 | 0.31 | 0.64 | 19.98 ^ns^ | 0.58* | 4 | 0.67 | 3.14 | 1.78 |
| Semi-invaded | 10 | 0.30 | 0.60 | 10.91 ^ns^ | 0.64* | 4 | 0.85 | 2.19 | 1.76 |
| Invaded | 9 | 0.36 | 0.60 | 15.94 ^ns^ | 0.62* | 4 | 0.82 | 2.06 | 1.81 |
| COMBINED | 20 | 0.22 | 0.60 | 16.65 ^ns^ | 0.65* | 6 | 0.74 | 3.42 | 2.02 |
| **98 % DNA similarity level** | | | | | | | | | |
| Uninvaded | 11 | 0.33 | 0.63 | 22.31 ^ns^ | 0.53* | 4 | 0.68 | 2.78 | 2.24 |
| Semi-invaded | 7 | 0.34 | 0.59 | 9.68 ^ns^ | 0.60* | 3 | 0.89 | 1.68 | 1.98 |
| Invaded | 6 | 0.40 | 0.60 | 12 ^ns^ | 0.59* | 3 | 0.86 | 1.55 | 2.04 |
| COMBINED | 16 | 0.23 | 0.57 | 15.88 ^ns^ | 0.59* | 5 | 0.73 | 2.31 | 2.49 |
| **95 % DNA similarity level** | | | | | | | | | |
| Uninvaded | 9 | 0.38 | 0.64 | 32.61 ^ns^ | 0.46* | 4 | 0.65 | 2.54 | 2.71 |
| Semi-invaded | 5 | 0.48 | 0.66 | 24.17 ^ns^ | 0.40* | 2 | 0.70 | 1.68 | 3.02 |
| Invaded | 4 | 0.55 | 0.66 | 27.08 ^ns^ | 0.41* | 2 | 0.73 | 1.50 | 3.05 |
| COMBINED | 12 | 0.28 | 0.59 | 28.35 ^ns^ | 0.47* | 3 | 0.61 | 2.19 | 3.62 |

p<0.05 from z-scores. ns – indicates no significant nestedness from randomization tests.

**File 3**: Species level network metrics for plants and bacteria at the genotype and 98% similarity levels. Means ± standard deviations of degree, effective partners and specialization (d’) across species at each site along the invasion gradient are shown. F and Χ^2^ statistics are for comparisons of metrics across sites.

|  | Degree | | Effective partners | | Specialisation (d’) | |
| --- | --- | --- | --- | --- | --- | --- |
|  | Genotype | 98% | Genotype | 98% | Genotype | 98% |
| **Plants** |  |  |  |  |  |  |
| Uninvaded | 5.2 ± 2.8 | 3.6 ± 1.9 | 4.2 ± 1.5 | 2.8 ± 1.4 | 0.91 ± 0.11 | 0.55 ± 0.23 |
| Semi invaded | 4.8 ± 1.8 | 2.4 ± 0.5 | 3.7 ± 1.5 | 1.7 ± 0.4 | 0.89 ± 0.12 | 0.61 ± 0.21 |
| Heavily invaded | 4.6 ± 1.5 | 2.4 ± 0.5 | 3.3 ± 1.4 | 1.6 ±0.3 | 0.85 ± 0.14 | 0.59 ± 0.22 |
|  | F_2,12_=0.11 ^ns^ | F_2,12_=1.64 ^ns^ | F_2,12_=0.43 ^ns^ | F_2,12_=3.23 ^ns^ | F_2,12_=0.78 ^ns^ | F_2,12_=0.10 ^ns^ |
| **Bacteria** |  |  |  |  |  |  |
| Uninvaded | 1.1 ± 0.3 | 1.6 ± 1.0 | 1.1 ± 0.3 | 1.4 ± 0.8 | 0.32 ± 0.25 | 0.35 ± 0.23 |
| Semi invaded | 1.2 ± 0.4 | 1.7 ± 1.1 | 1.1 ± 0.3 | 1.5 ± 0.7 | 0.36 ± 0.24 | 0.54 ± 0.25 |
| Heavily invaded | 1.4 ± 0.6 | 2.0 ± 1.1 | 1.2 ± 0.4 | 1.7 ± 0.6 | 0.38 ± 0.27 | 0.54 ± 0.33 |
|  | Χ^2^=3.1 ^ns^ | Χ^2^=1.5 ^ns^ | Χ^2^=3.1 ^ns^ | Χ^2^=1.5 ^ns^ | Χ^2^=0.9 ^ns^ | Χ^2^=4.2 ^ns^ |


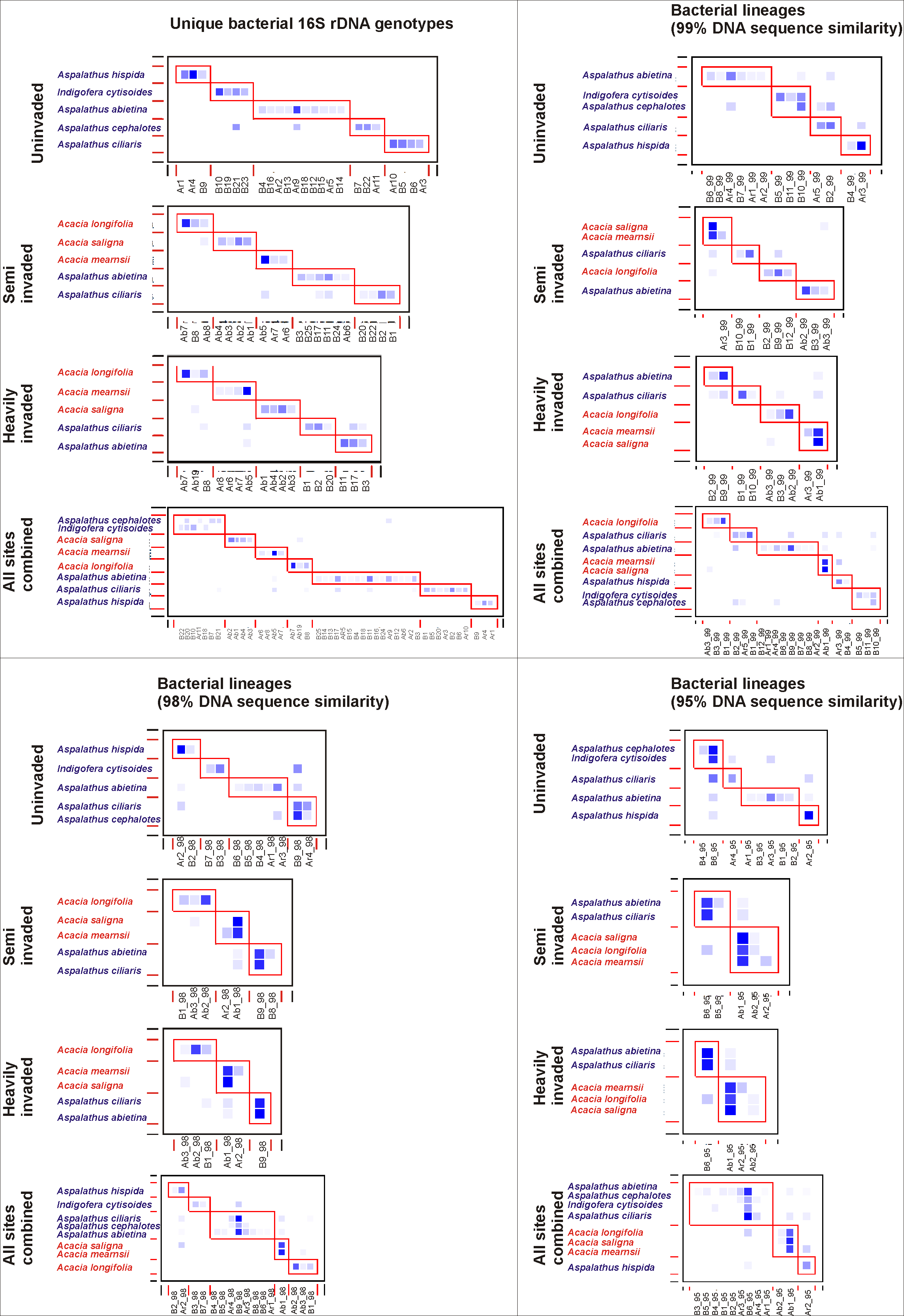


**File 4.** Interaction networks representing legume-rhizobia interactions across a gradient of *Acacia* invasion (uninvaded, semi invaded and heavily invaded) as well as for the combined dataset for hierarchically delineated rhizobial taxa (genotype, 99%, 98% and 95% sequence similarity levels). Rows represent plant taxa (invasives = red; natives = blue). Columns represent nodule associated bacterial taxa (B – betaproteobacteria in the genus *Burkholderia*, Ar – alphaproteobacteria in the fast-growing*Rhizobium* clade (Fig 1, node B), Ab – alphaproteobacteria in the slow-growing *Bradyrhizobium* clade (Fig. 1, node A)). The frequency of species interactions is represented by increasing intensity of blue and red boxes represent modules identified by the weighted modularity approach of Dormann and Strauss (2014).
